# Unilateral interlaminar fenestration on the convex side provides a reliable access for intrathecal administration of nusinersen in spinal muscular atrophy: a retrospective study

**DOI:** 10.1186/s13023-023-02972-3

**Published:** 2023-11-29

**Authors:** Zhen Wang, Erwei Feng, Yang Jiao, Junduo Zhao, Xin Chen, Haozhi Zhang, Jinqian Liang, Zheng Li, Xulei Cui, Weiyun Chen, Jianxiong Shen

**Affiliations:** 1 Department of Orthopedics, Peking Union Medical College Hospital, Peking Union Medical College and Chinese Academy of Medical Sciences, Beijing, People’s Republic of China; 2 Department of Anesthesiology, State Key Laboratory of Complex Severe and Rare Diseases, Peking Union Medical College Hospital, Peking Union Medical College and Chinese Academy of Medical Sciences, Beijing, People’s Republic of China

**Keywords:** Spinal muscular atrophy, Scoliosis, Posterior spinal fusion, Interlaminar fenestration, Nusinersen administration, Intrathecal injection, Ultrasound guidance

## Abstract

**Background:**

As the first gene therapy for spinal muscular atrophy (SMA), nusinersen is supposed to be administrated via intrathecal injection regularly for a lifetime. However, for SMA patients with severe spinal deformities, bony fusion following posterior spinal instrumentation sets great obstacles for the application of nusinersen. Therefore, efforts have been devoted to the exploration of appropriate approach for nusinersen administration. This study aims to evaluate the safety and reliability of unilateral interlaminar fenestration on the convex side during spinal fusion surgery for intrathecal nusinersen injection in SMA.

**Results:**

SMA patients receiving posterior spinal fusion and interlaminar fenestration in Peking Union Medical College Hospital from January 2020 to October 2021 were retrospectively analyzed. 13 patients were included. Of the 13 patients, 10 were classified into SMA type II and 3 into SMA type III. Distal fusion to pelvis was undertaken in 11 patients; while L5 was selected as the lowest instrumented vertebra in the other 2 patients. All patients received interlaminar fenestration on the convex side only with an area of about 15 mm × 20 mm. Fenestration at L2–L3 level was performed in 6 patients; while L3–L4 level was selected for windowing in the remaining 7 patients. 9 of the 13 patients received lumbar puncture and intrathecal nusinersen administration during the 1-year follow-up, with an accumulative total of 50 times. All injections were performed successfully under ultrasound guidance, with no one transferred to radiographic assistance. No severe complications occurred after injection.

**Conclusions:**

In SMA with severe scoliosis planning to receive posterior spinal fusion, unilateral lumbar interlaminar fenestration on the convex side provides a feasible and reliable access for intrathecal nusinersen administration after surgery.

## Introduction

Spinal muscular atrophy (SMA) is the most common genetic factor for infant death, with an incidence of 1/12,000 in neonates [1]. Apart from motor and respiratory dysfunction, muscular weakness in SMA may also lead to skeletal deformity. The prevalence of scoliosis in SMA patients exceeds 60% [2]. Scoliosis in SMA is characterized by early onset and rapid progression. Severe scoliosis and kyphosis in SMA bring about thoracic deformity, pelvic obliquity and cervical rotation, and thus reduce life quality and expectancy [3]. Bracing is not well-tolerated for SMA patients and incapable of preventing the progression of deformities [4]. Therefore, surgical treatment has become an important means to correct spinal deformities and improve quality of life in SMA patients. However, either growing rod or spinal fusion does not prevent the deterioration of muscle weakness and atrophy.

As the first FDA-approved gene therapy for SMA, nusinersen promotes the expression of full-length functional SMN protein through interfering with the splicing of exon-7 in *SMN2* transcripts [5, 6]. Previous studies demonstrated that nusinersen prolongs survival of patients with SMA type I and improves motor function of patients with SMA type II [7, 8]. Besides, nusinersen also improves respiratory muscle strength and delays respiratory impairment in SMA patients [9]. Nowadays, nusinersen has been applied to the treatment of SMA of all types. As an antisense oligonucleotide, nusinersen is vulnerable to degradation and unable to penetrate the blood–brain barrier [10]. Therefore, it is required to be administrated via intrathecal injection periodically for a lifetime. For SMA patients with complex spinal deformities, posterior spinal fusion (PSF) brings about osseous fusion of posterior elements, and thus set obstacles for lumbar puncture via interspinous or interlaminar approach [11]. To preserve intrathecal access for SMA patients, Konigsberg and colleagues [12] suggested skipping one or more intervertebral levels at the thoracolumbar junction during PSF. However, this strategy retains the mobility of segments preserved simultaneously, which may increase the risks of correction loss and implant failure in long-term follow-up. Recently, intrathecal catheter with a port placed subcutaneously was applied for the administration of nusinersen. However, the medium- and long-term mechanical complications of catheter placement and the risks of catheter-related infections deserve vigilance [13–15]. In a word, how to achieve intrathecal nusinersen administration safely and effectively in SMA patients with severe spinal deformities requiring spinal fusion remains a challenge.

This study aims to evaluate the effectiveness and safety of unilateral lumbar interlaminar fenestration on the convex side for intrathecal administration of nusinersen in SMA patients treated with PSF.

## Methods

### Subjects

After the approval of the institutional review board, medical records of SMA patients from January 2020 to October 2021 were reviewed. Inclusion criteria included: (1) SMA with definite genetic diagnosis. (2) Treated with PSF and unilateral interlaminar fenestration (UILF) simultaneously. (3) Follow-up for at least 1 year after surgery. Exclusion criteria were as follows: (1) PSF without UILF was performed. (2) Insufficient follow-up. Informed consent was obtained from adult patients or legal guardians.

### Data collection and clinical evaluation

Demographic data, including gender and age of surgery, were collected. Clinical classification was performed by a senior neurologist majored in neuromuscular disorders. Main curve Cobb angle (MCCA), pelvic obliquity (PO) and kyphosis were measured preoperatively and postoperatively. Surgical details, including fusion range and the location of fenestration, were recorded. Frequency of nusinersen administration, imaging methods for the guidance of intrathecal injection and complications after lumbar puncture were analyzed.

### Surgical details

**Fig. 1:**
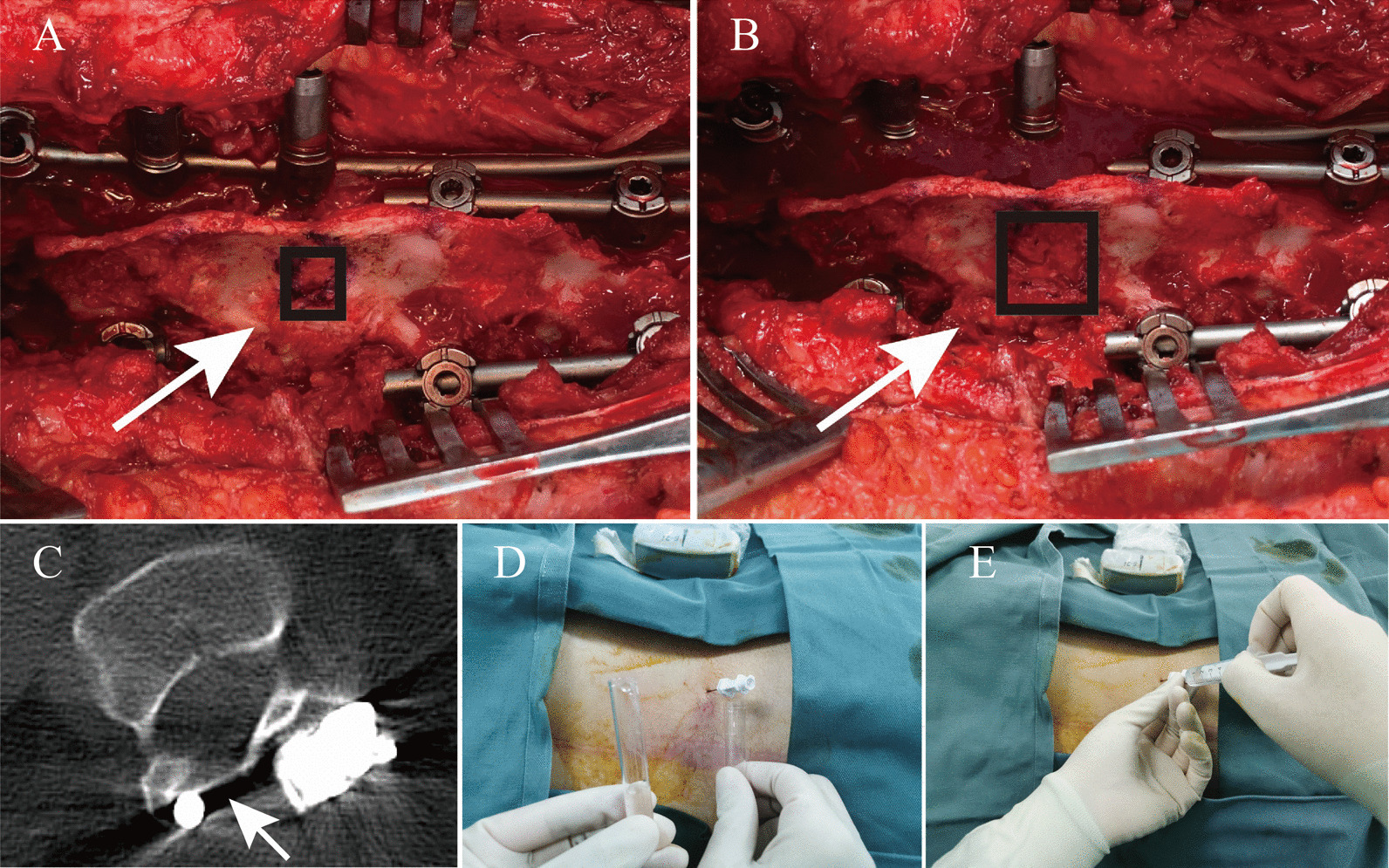
Unilateral fenestration at L3–L4 interval on the convex side for nusinersen administration after spinal fusion. **A** Narrow interlaminar space (black box indicated by white arrow) before fenestration. **B** Enlarged interlaminar space (black box indicated by white arrow) after fenestration. **C** Enlarged interlaminar space without ossification and closure (indicated by the white arrow) confirmed by axial computed tomography scan 3 months after surgery. **D** Successful lumbar puncture via fenestrated interlaminar space under ultrasound guidance. **E** Intrathecal nusinersen injection via fenestrated interlaminar space

After general anesthesia, patients were placed in prone position. Posterior median incision was adopted. Subperiosteal dissection of paravertebral muscles was performed to the transverse process root areas. Pedicle screws of suitable length and diameter were instrumented. Then multiple routine maneuvers and techniques were applied to achieve apical region correction and global balance. Fluoroscopy was performed to confirm corrective effect. After the achievement of satisfactory correction, interlaminar space at L2–L3 or L3–L4 was explored (Fig. 1A). UILF on the convex side with a window of approximately 15 mm × 20 mm was performed (Fig. 1B). Bone wax was used to seal the surface of the bone and the window was covered by gelatin sponge. Then laminae outside the window were decorticated and mixed bone graft was applied for fusion.

### Technotes for lumbar puncture and intrathecal administration

Patients lied in lateral position and the back was exposed. Ultrasound scan of the lumbar spine was performed with a low-frequency curvilinear transducer to localize the L2–L3 or L3–L4 segment. Then ultrasonic probe was placed 2–3 cm deviated from the midline on the convex side, in parallel with the longitudinal axis of the body. Titanium rod with strong echo was identified. Then paramedian sagittal scan was performed from the rod to midline. The direction of the probe was adjusted gradually until the high echo of bone interrupted and ultrasound beam entered spinal canal through the window of laminae interval space. Then the posterior dura and anterior complex with hyperechoic signal were detected and position for puncture were marked. After sterile preparation and local anesthesia, a 22 gauge, 10-cm echogenic needle with a short-bevel tip and stylet was penetrated layer by layer via the window to the subdural space under real-time ultrasonic monitoring. Then the stylet was removed and 5 mL of cerebrospinal fluid was collected for cytologic and biochemical examinations. Nusinersen of equal volume (5 mL/12 mg) was administrated. After the removal of the needle, patients were placed in supine position and observed for 1 h. Imaging measures, including fluoroscopy and computed tomography, were used as an alternative method when ultrasound-guided puncture failed.

## Results

### Demographic data, clinical classification, surgical details and radiographic parameters

**Fig. 2:**
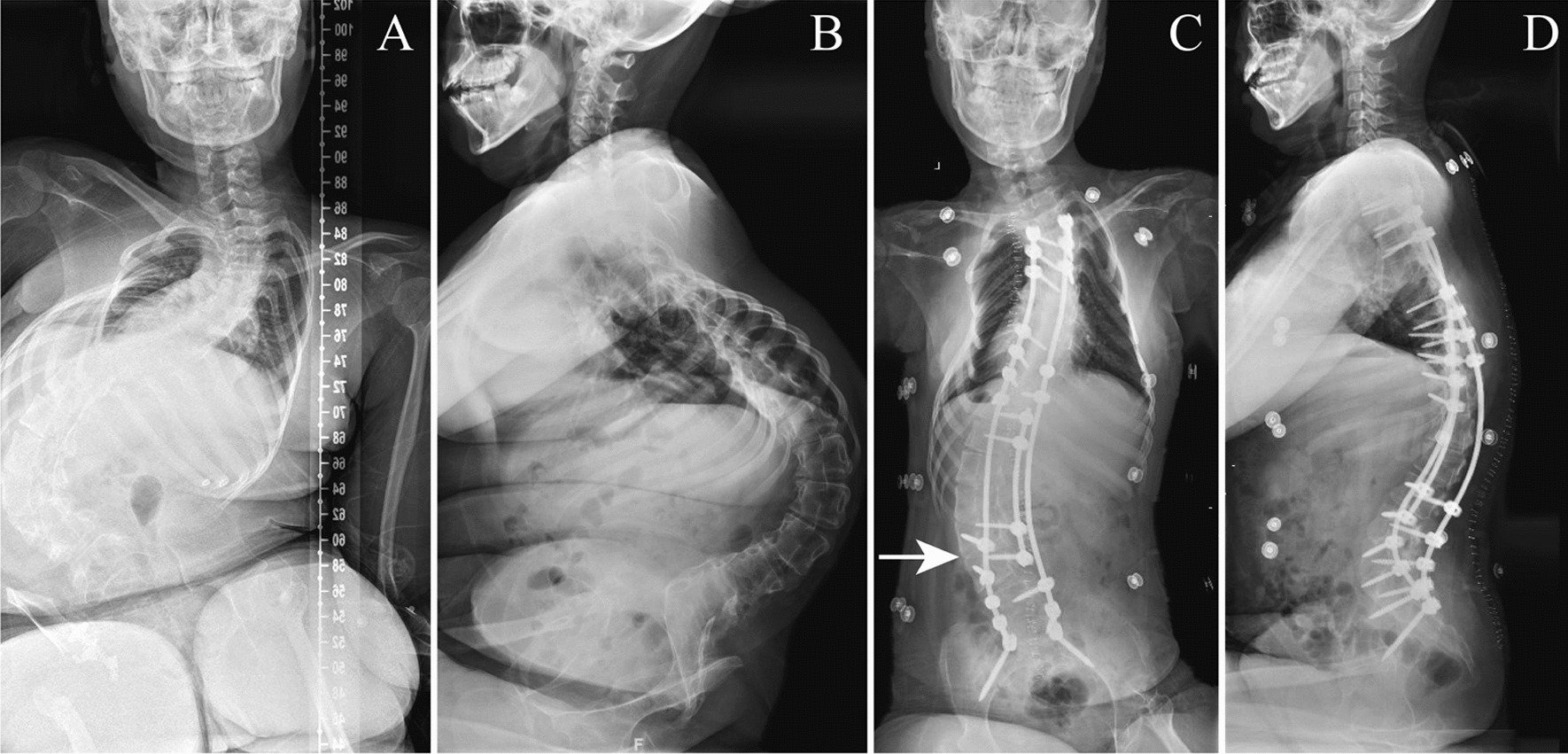
A 20-year-old female with SMA type II underwent posterior spinal fusion from T3 to pelvis. **A**, **B** Preoperative radiographs demonstrate a Cobb angle of 115.3° (T9–L4), pelvic obliquity of 28.9° and kyphosis of 102.4° (T4–L5). **C**, **D** Postoperative radiographs demonstrate good surgical outcomes with Cobb angle of 52.6° (T9–L4), pelvic obliquity of 11.6° and kyphosis of 21.3° (T4–L5). Unilateral interlaminar fenestration at L3–L4 level on the convex side (indicated by the white arrow) was performed during spinal fusion

A total of 13 SMA patients (5 male; 8 female) treated with PSF and UILF were enrolled (Table 1). Age at surgery ranged from 11 to 28 years old. Of the 13 patients, 10 were classified into SMA type II; while the remaining 3 into type III. Sacroiliac fixation with sacral-2-alar-iliac (S2AI) screws was applied in 11 patients. Distal fusion to L5 was undertaken in the other 2 patients. Fenestration at L2–L3 interspace was conducted in 6 patients; while L3–L4 interspace was selected for windowing in the remaining 7 patients. Preoperatively, MCCA and kyphotic angle were 119.2° ± 25.6° and 119.7° ± 28.3°, respectively. Postoperative MCCA and kyphotic angle were corrected to 56.6° ± 18.2° and 42.9° ± 17.6°, respectively (Fig. 2). Besides, PO was corrected from 35.0° ± 18.1° before surgery to 14.5° ± 14.0° after surgery (Table 2).

**Table 1:** Information of lumbar fenestration and nusinersen administration in SMA patients with spinal fixation

No	Gender	Age of surgery (y)	Clinical classification	Fusion range	Level of fenestration	Times of injection	Guiding methods (ultrasound/X-ray/CT)	Interval between surgery and first injection (m)	Types of complications	Times of complications
1	Male	26	II	T3-pelvis	L3/L4	5	Ultrasound	22	N	0
2	Female	17	II	T2-pelvis	L3/L4	0	–	–	–	–
3	Female	13	III	T3-pelvis	L3/L4	0	–	–	–	–
4	Female	12	II	T3-pelvis	L2/L3	9	Ultrasound	3	N	0
5	Male	12	II	T2-pelvis	L2/L3	0	–	–	–	–
6	Female	11	II	T3-pelvis	L2/L3	6	Ultrasound	11	Headache	3
7	Male	18	III	T3-pelvis	L2/L3	5	Ultrasound	13	Headache	2
8	Male	28	II	T6–L5	L3/L4	4	Ultrasound	18	N	0
9	Female	14	II	T3-pelvis	L3/L4	5	Ultrasound	12	N	0
10	Male	15	II	T3-pelvis	L2/L3	4	Ultrasound	15	Headache and nausea	2
11	Male	11	II	T4–L5	L2/L3	6	Ultrasound	3	N	0
12	Female	20	III	T4-pelvis	L3/L4	6	Ultrasound	5	Vertigo	1
13	Female	20	II	T3-pelvis	L3/L4	0	–	–	–	–

*SMA* Spinal muscular atrophy, *CT* Computed tomography

**Table 2:** Radiographic parameters before surgery and after surgery in SMA patients receiving posterior spinal fusion and lumbar fenestration

	Preoperative	Postoperative	*P* value
MCCA (°)	119.2 ± 25.6	56.6 ± 18.2	0.000
Kyphosis (°)	119.7 ± 28.3	42.9 ± 17.6	0.000
PO (°)	35.0 ± 18.1	14.5 ± 14.0	0.000

All values are described as the mean and standard deviation. Comparison between preoperative parameters and postoperative parameters was performed using paired *t* test

*SMA* Spinal muscular atrophy, *MCCA* Main curve Cobb angle, *PO* Pelvic obliquity

### Lumbar puncture and intrathecal administration

Before manipulation, wound healing was evaluated. Blood routine, erythrocyte sedimentation rate and C-reaction protein, were also detected to rule out wound infection. No wound complications and remarkable abnormalities in blood tests were observed. Of the 13 patients with extended windowing, 9 accepted lumbar puncture and nusinersen injection during follow-up, with medication ranging from 4 to 9 times (an average of 5.6 ± 1.5 times). An accumulative total of 50 times administration has been accomplished. Preoperative CT scans were performed merely before the first administration in the first patient receiving nusinersen administration to ensure the unobstruction of the access (Fig. 1C). All subsequent procedures were successfully performed under ultrasound guidance, without the assistance of fluoroscopy or CT scans (Fig. 1D, E). Intervals between spinal fusion surgery and the first intrathecal administration varied from 3 to 22 months (an average of 11.3 ± 6.7 months).

### Complications

Minor complications were recorded in 4 of the 9 patients receiving nusinersen injection. 1 patient with SMA type II experienced headache and nausea at the first and third administration. Headache was complained in 1 patient with SMA type II at the first, third and fifth injections. Another patient with SMA type III had headache at the first 2 injections. Vertigo occurred at the first injection in a SMA type II patient. Above symptoms relieved spontaneously after rest in supine position. No severe complications occurred.

## Discussion

The introduction of nusinersen brings a new era to the treatment of SMA. However, how to deliver nusinersen safely and effectively has become a novel challenge in clinic. Severe pelvic obliquity in SMA necessitates spinopelvic fixation in most cases [16]. Fusion of posterior elements following spinal correction erects a definite roadblock for lumbar puncture and intrathecal administration via conventional L3–L4 or L4–L5 interlaminar space. In addition to interspinous and interlaminar approaches, transforaminal approach is also an important way to administrate nusinersen in SMA patients with complex spinal anatomy or spinal fusion [11]. However, various complications have been reported during transforaminal nusinersen injection, such as dural injury, epidural hematoma and subarachnoid hemorrhage [17–19]. Besides, preoperative evaluation and intraoperative guidance via radiographic methods are usually required for transforaminal lumbar puncture [20]. In view of the necessity of lifetime use of nusinersen, extra radiation exposure induced by repeated administration does not go unnoticed. Cervical puncture via atlantoaxial or atlantooccipital approach was also reported to deliver nusinersen under ultrasonic or fluoroscopic guidance [21, 22]. However, above administration approaches imply a high risk of injuries in adjacent structures, such as spinal cord, vertebral artery and posterior inferior cerebellar artery. Apparently, cervical puncture requires extensive anatomy knowledge and thorough experience, which might restrict the use of these approaches for nusinersen administration. Therefore, a reliable access for long-term nusinersen administration is supposed to be taken into account during the surgical management of spinal deformities in SMA.

Multiple surgical strategies have been reported to provide a convenient and effective drug delivery channels for SMA patients with severe spinal deformities, who are planning to have or have already undergone PSF. Cordts et al. [18] utilized translaminar drilling to create a corridor for intrathecal administration. However, interventions via the new osseous canal were accomplished with the assistance of CT-guidance, which may increase the burden of radiation exposure. Moreover, in view of the 4-month administration interval at the maintaining stage of nusinersen, whether the canal will be ossified and closed requires long-term observation. Intrathecal catheter was also applied for the administration of nusinersen. Generally, catheter was implanted via hemilaminectomy or drilling with a port placed subcutaneously [13, 23, 24]. This method not only avoid radiation exposure but also reduce the usage of narcotics. The simplicity and practicality of catheter-assisted nusinersen administration enables intrathecal injection in outpatient department. However, the possibility of mechanical compression, occlusion, separation and leakage may limit the use of catheter. In addition, the catheter interferes with intraspinal structures and increases the risks of central nervous system infection [13–15].

As reported, higher termination of dural sac in some SMA patients may limited the use of L5–S1 interspace for lumbar puncture [25]. In our cohort, attempt has been made to administrate nusinersen intrathecally via L5–S1 interval in 1 of the 2 patients with L5 as lowest instrumented vertebra but in vain. Therefore, UILF was also undertaken in patients distally fused to L5. Similar to fenestration in this study, Labianca and Weinstein [4] reported a laminotomy at the L3/L4 level for nusinersen administration in SMA patients. Fat grafting was performed to prevent the closure of the window. Besides, hemoclips was used as radiographic references to localize the window. Machida and colleagues [26] combined L3 laminectomy with PSF to create an access for nusinersen administration. A transverse connector was placed at the level of L3 laminectomy as a radiographic marker. Particularly, decortication of the bilateral facet joints at L2/L3 and L3/L4 levels and the subsequent autograft was conducted to obtain bony fusion at the laminectomy level. In addition to hemoclips and transverse connector, circumferential bone screws were also used to localize the fenestrated intralaminar interval [27]. Intrathecal nusinersen administration was accomplished under fluoroscopic guidance in all above 3 studies. Compared with these studies, UILF on the convex side with an area of 15 mm × 20 mm in our cohort provided an adequate access for the reliable and repeatable nusinersen injection, with minimal intervention with spinal fusion. Spontaneous fusion of the fenestrated window was prevented, confirmed by the successful administration of nusinersen in all procedures. Besides, markers for radiation were placed in none of the patients. All injections were accomplished under the guidance of ultrasound, with no one transferred to radiographic assistance. On this basis, the strategy we put forward may avoid unnecessary implants and repeated radiation exposure simultaneously. To our knowledge, little study implied the interval between PSF and the first nusinersen injection after surgery. Herein, we demonstrated the feasibility and safety of intrathecal administration 3 month after surgery. Besides, comprehensive evaluation of wound healing and blood tests is recommended to rule out the possibility of infection. Noteworthily, 4 of the 9 patients receiving nusinersen treatment exhibited minor complications associated with cerebrospinal fluid leakage. Now that it may help to avoid postoperative epidural adhesions and spinal fluid leak after lumbar puncture, fat grafting may be applied during surgery in the future.

There are some limitations in this study. First, the sample size is relatively small. Thereby, the incidence of complications might be estimated inaccurately. Second, the follow-up period is not sufficient to determine the incidence of implant breakage. Third, comparison of the efficacy and the incidence of complications of nusinersen injection between the UILF and intrathecal catheter with a port is in lack. In the future, study with longer follow-up, larger samples and a concurrent control group will be performed to validate the effectiveness of UILF on the convex side for nusinersen administration in SMA.

## Conclusions

This study introduced a novel surgical strategy which suggests additional UILF on the convex side during PSF in SMA patients for intrathecal administration postoperatively. Lumbar puncture via fenestrated interspace under ultrasound guidance permits the safe and effective injection of nusinersen after surgery.

## Data Availability

The datasets generated and analysed during the current study are available in the Figshare repository, https://figshare.com/articles/dataset/_b_Unilateral_interlaminar_fenestration_on_the_convex_side_provides_a_reliable_access_for_intrathecal_administration_of_nusinersen_in_spinal_muscular_atrophy_a_retrospective_study_b_/24250339.

## Abbreviations

SMA: Spinal muscular atrophy
PSF: Posterior spinal fusion
UILF: Unilateral interlaminar fenestration
MCCA: Main curve Cobb angle
PO: Pelvic obliquity
S2AI: Sacral-2-alar-iliac
